# Supplementary material for: Long-wave infrared photothermoelectric detectors with ultrahigh polarization sensitivity
Source: Nat Commun. 2023 Jun 9;14:3421. doi: 10.1038/s41467-023-39071-7 (PMC10256712; doi:10.1038/s41467-023-39071-7)
Supplement: Supplementary file 2 — Supplementary Information [file 41467_2023_39071_MOESM2_ESM.pdf]

**Supplementary Information**

**for**

# Long-wave infrared photothermoelectric detectors with ultrahigh polarization sensitivity

Mingjin Dai[1†], Chongwu Wang[1†], Bo Qiang[1], Yuhao Jin[1], Ming Ye[1], Fakun Wang[1], Fangyuan Sun[1], Xuran Zhang[1], Yu Luo[1], and Qi Jie Wang[1,2]

[1]School of Electrical and Electronic Engineering, Nanyang Technological University, Singapore 639798, Singapore.

[2]Centre for Disruptive Photonic Technologies, School of Physical and Mathematical Sciences, Nanyang Technological University, Singapore 637371, Singapore.

[†]These authors contributed equally to this work.

Corresponding Author: qjwang@ntu.edu.sg; luoyu@ntu.edu.sg

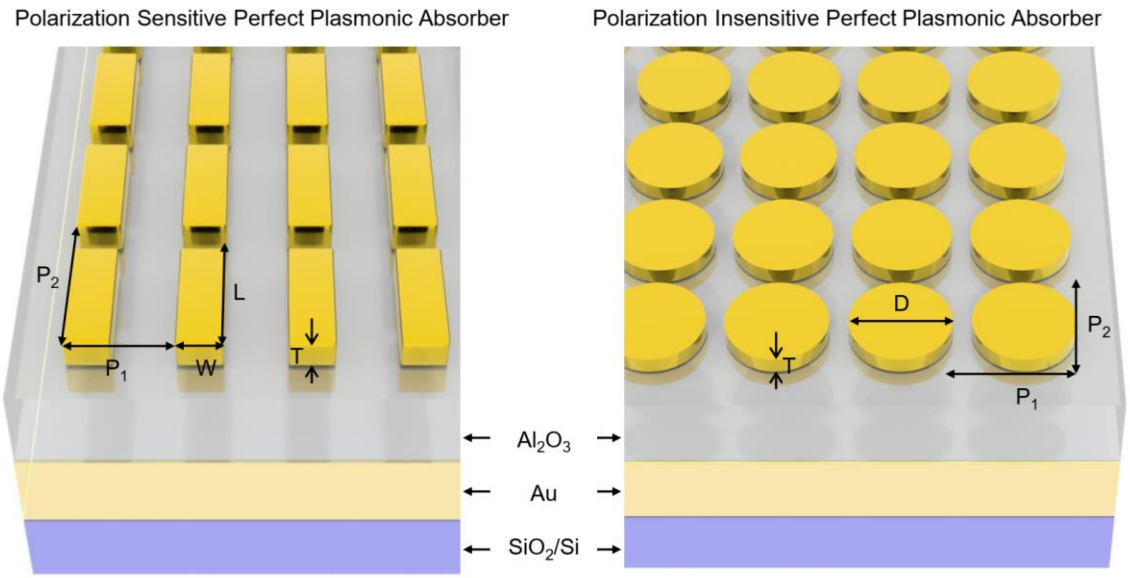


**Supplementary Figure 1: Structure diagram of the polarization sensitive/insensitive perfect plasmonic absorber with structure parameters.** $P_1$ and $P_2$ indicate the period length along x and y direction. W and L indicate the width and length of the rectangle microstructure. D indicates the diameter of the micro-disk. T indicates the thickness of microstructures.

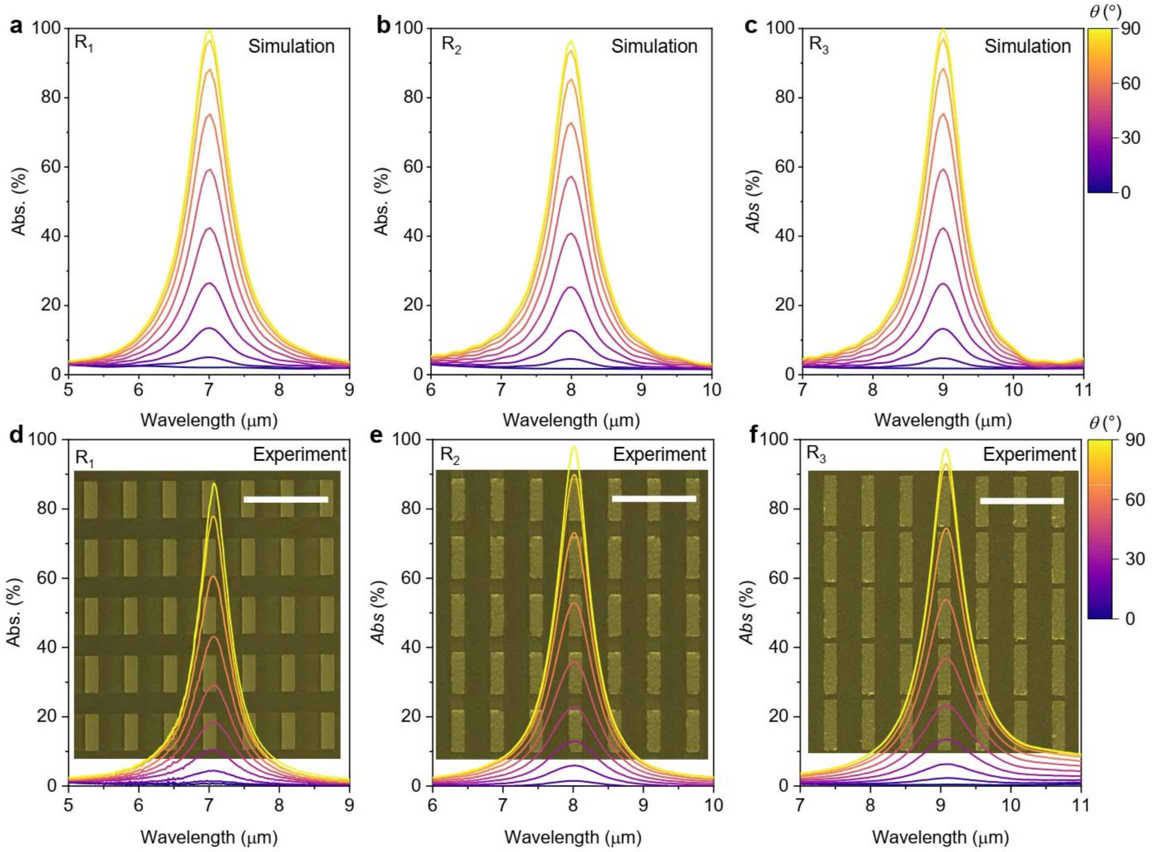


**Supplementary Figure 2: Simulated and experimental measured linear polarization dependent optical absorption spectra of perfect plasmonic absorbers. a-c**, Simulated linear polarization dependent optical absorption spectra of perfect plasmonic absorber $R_1$ (**a**) $R_2$ (**b**), and $R_3$ (**c**). **d-f**, Experimental measured optical linear polarization dependent optical absorption spectra of perfect plasmonic absorber $R_1$ (**d**), $R_2$ (**e**) $R_3$ (**f**). Insets: SEM images of corresponding perfect plasmonic absorbers. Scale bars: 5 μm.

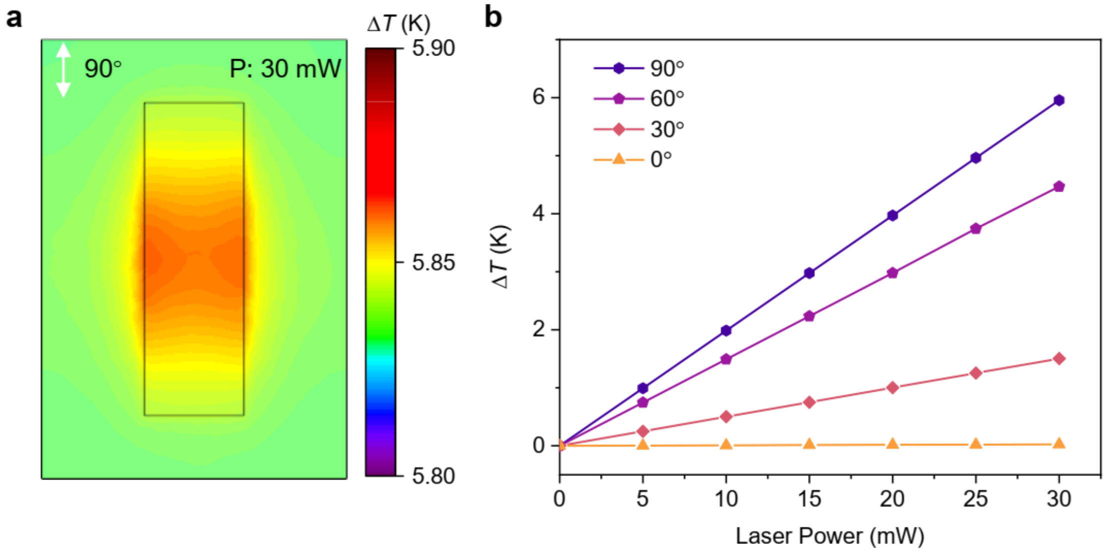


**Supplementary Figure 3: Simulation of the photothermal effect of the perfect plasmonic absorber. a**, Simulated temperature distribution in the Au microstructure under illumination with a linear polarization angle of 90° and a laser power of 30 mW. **b**, Laser power dependent temperature increase ($\Delta T$) for different polarization states.

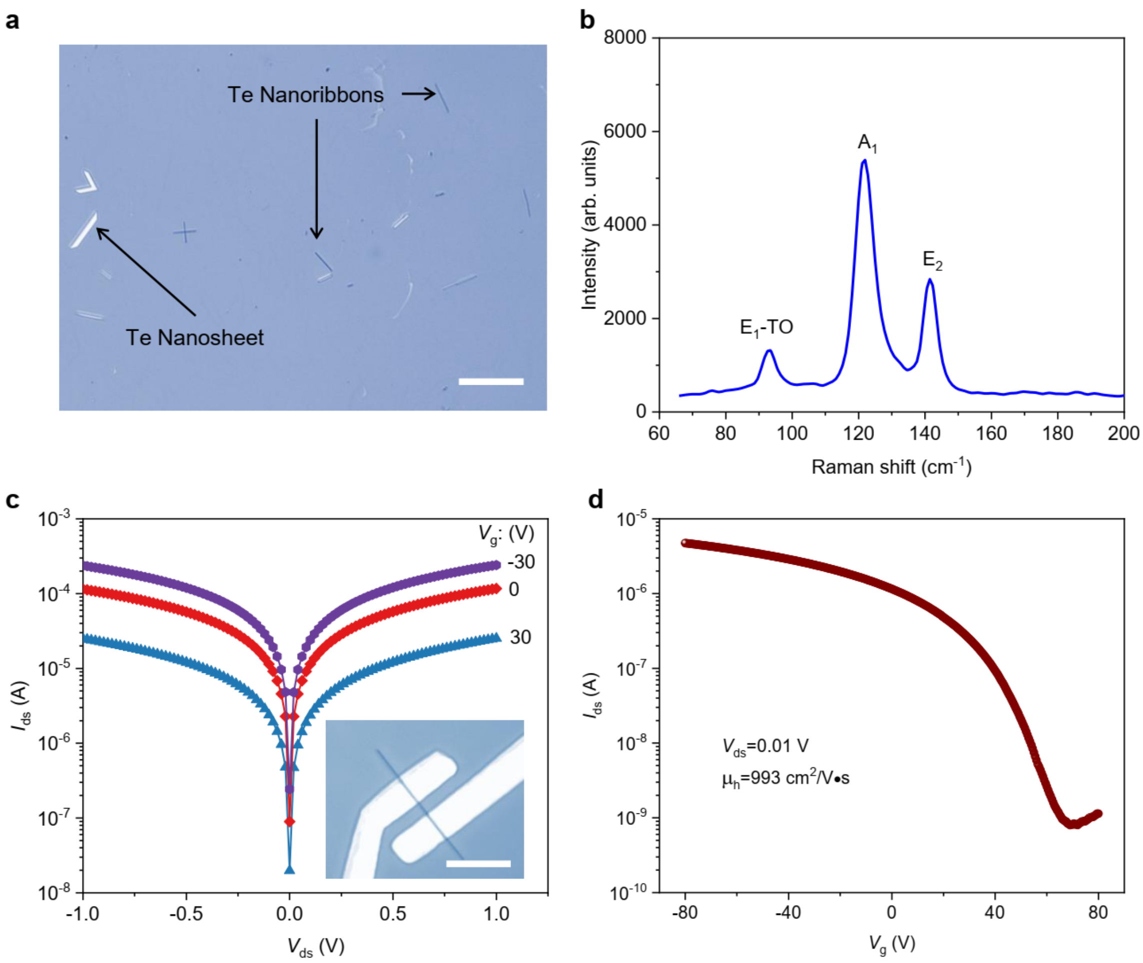


**Supplementary Figure 4: Characterization of as-synthesized Te nanoribbons.** **a**, A typical image of synthesized Te products transferred onto a $SiO_2$/Si substrate with 1D nanoribbon and 2D nanosheets. Scale bar: 20 µm. **b**, Raman spectrum of Te nanoribbon shows three modes localized at 93, 121, and 141 $cm^{-1}$, respectively. **c**, Output curves under different gate biases $V_g$. Inset shows the optical image of the 1D Te nanoribbon-based device. Scale bar: 10 µm. **d**, Transfer curve measured under a source-drain bias $V_{ds}$=0.01 V showing a p-type transport behavior with a hole mobility of 993 $cm^2$/V•s.

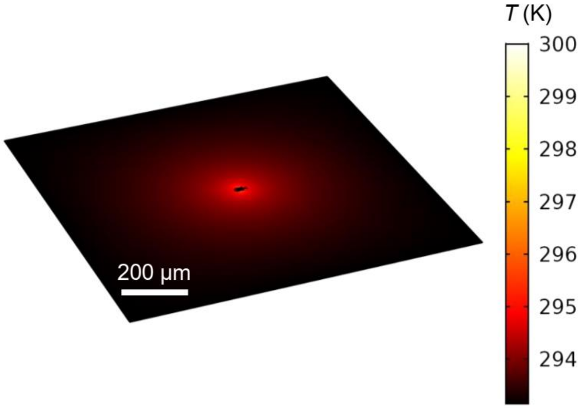


**Supplementary Figure 5: Simulation of the temperature distribution of the device in a large scale.** The boundary condition is set to be room-temperature (293 K) because of the large device scale (1×1 $mm^2$).

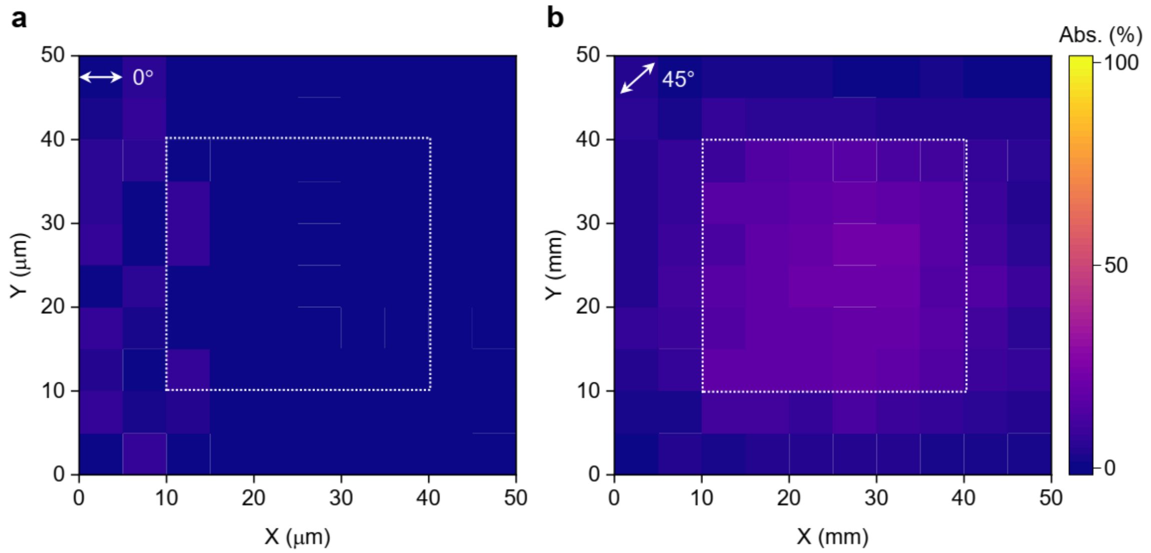


**Supplementary Figure 6: Polarization dependent absorption mapping of the perfect plasmonic absorber array with a finite-size of 30×30 μm$^2$. a**, **b**, The absorption mappings of the metamaterial for linear polarized light at wavelength of 8 μm with the polarization angle 0° (**a**) and 45° (**b**), respectively.

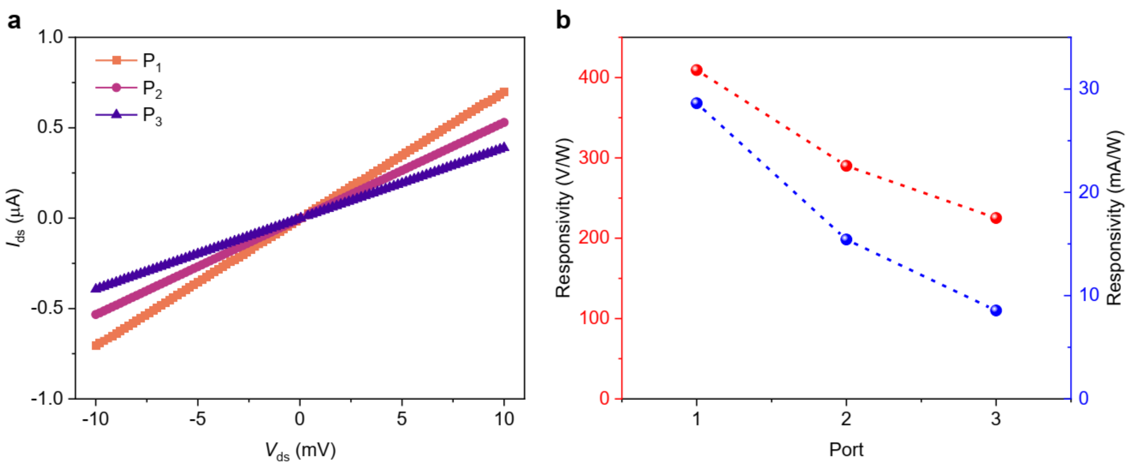


**Supplementary Figure 7: High responsivity of the devices with different channel lengths. a**, $I_{ds}$-$V_{ds}$ curves for different channel length in the device with three ports. **b**, The photovoltage and photocurrent responsivities for different ports as shown in Fig. 3 in the main text.

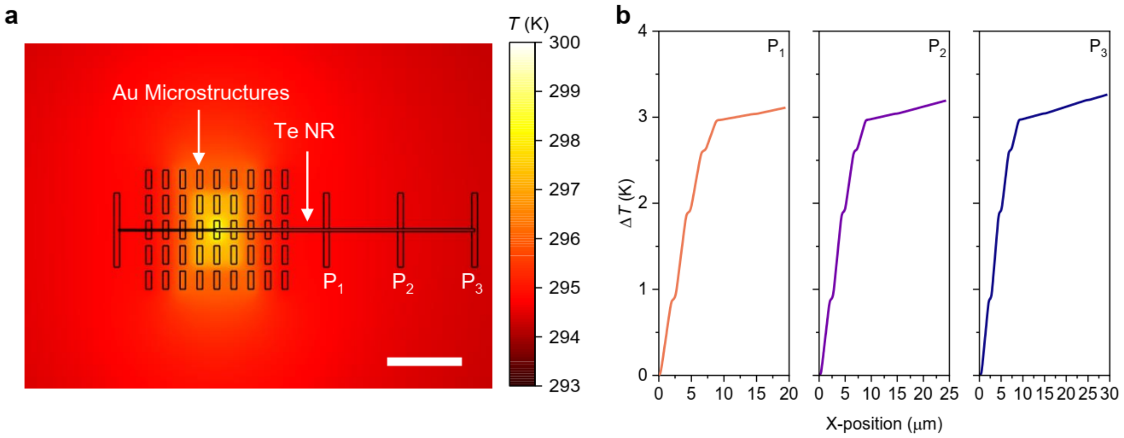


**Supplementary Figure 8: Simulation of the temperature distribution of the device with three ports. a**, Temperature distribution in the device with three ports. **b**, The temperature increasing profiles along the Te NW for different ports with a fixed incident laser power.

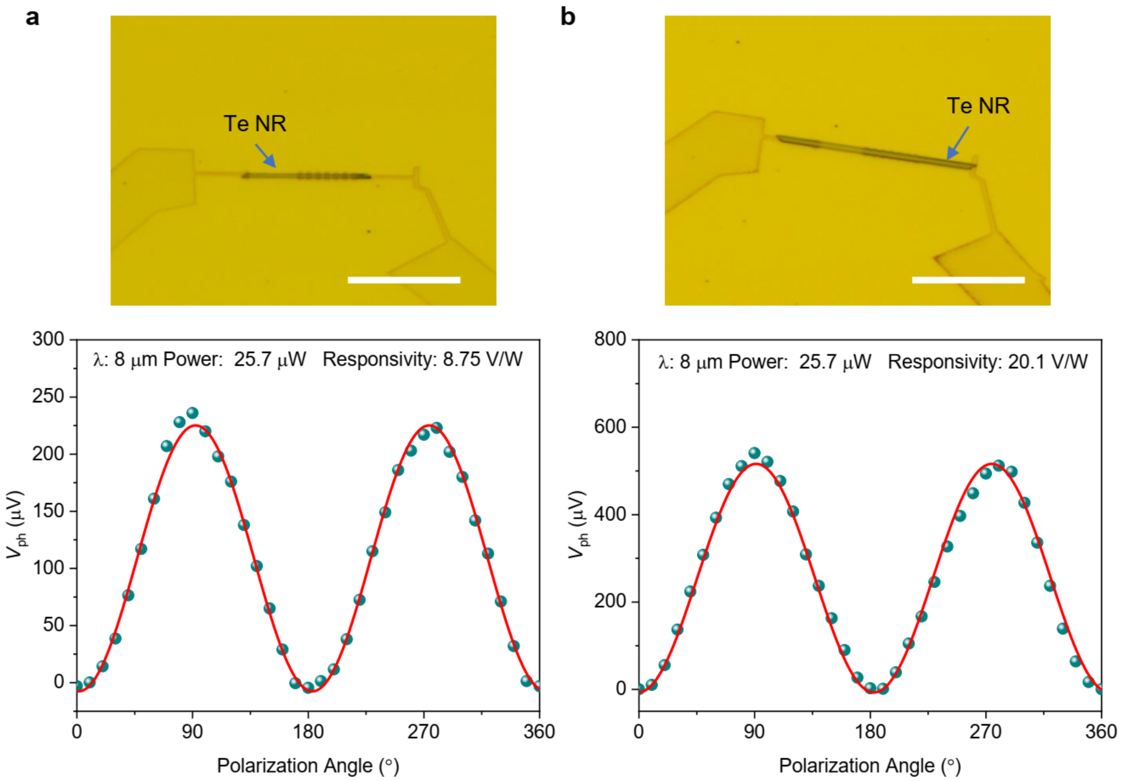


**Supplementary Figure 9: Polarization-sensitive photoresponses of the devices with only contacting meta-atoms. a, b,** Optical images (top) of the devices with only contacting meta-atoms and polarization-sensitive photoresponses (bottom). Scale bar: 30 μm. Dots are measured data, and red lines are fitting curves.

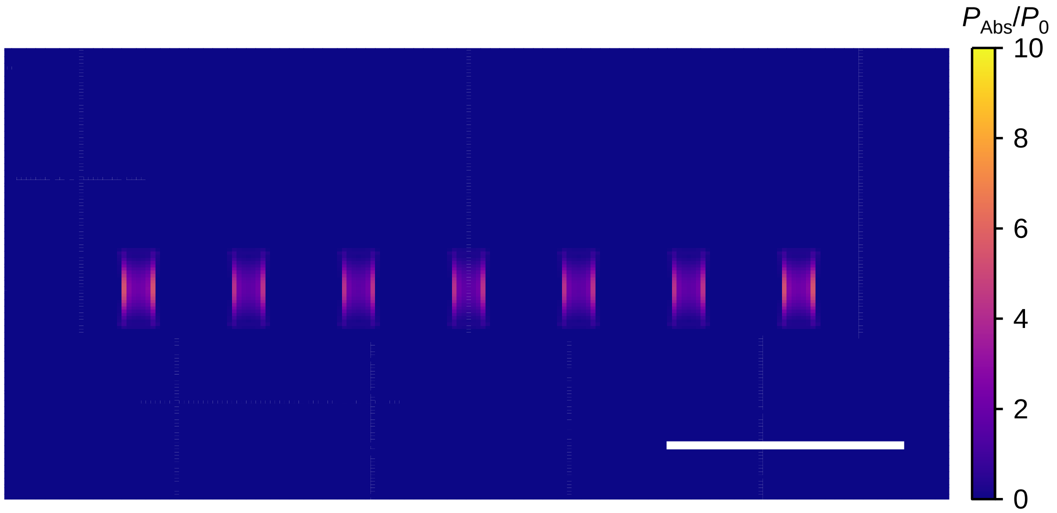


**Supplementary Figure 10: Simulated absorption power density of the contacting meta-atoms.** The absorption is relative lower than that of periodic metasurface owing to the finite-size effect. Scale bar: 5 μm.

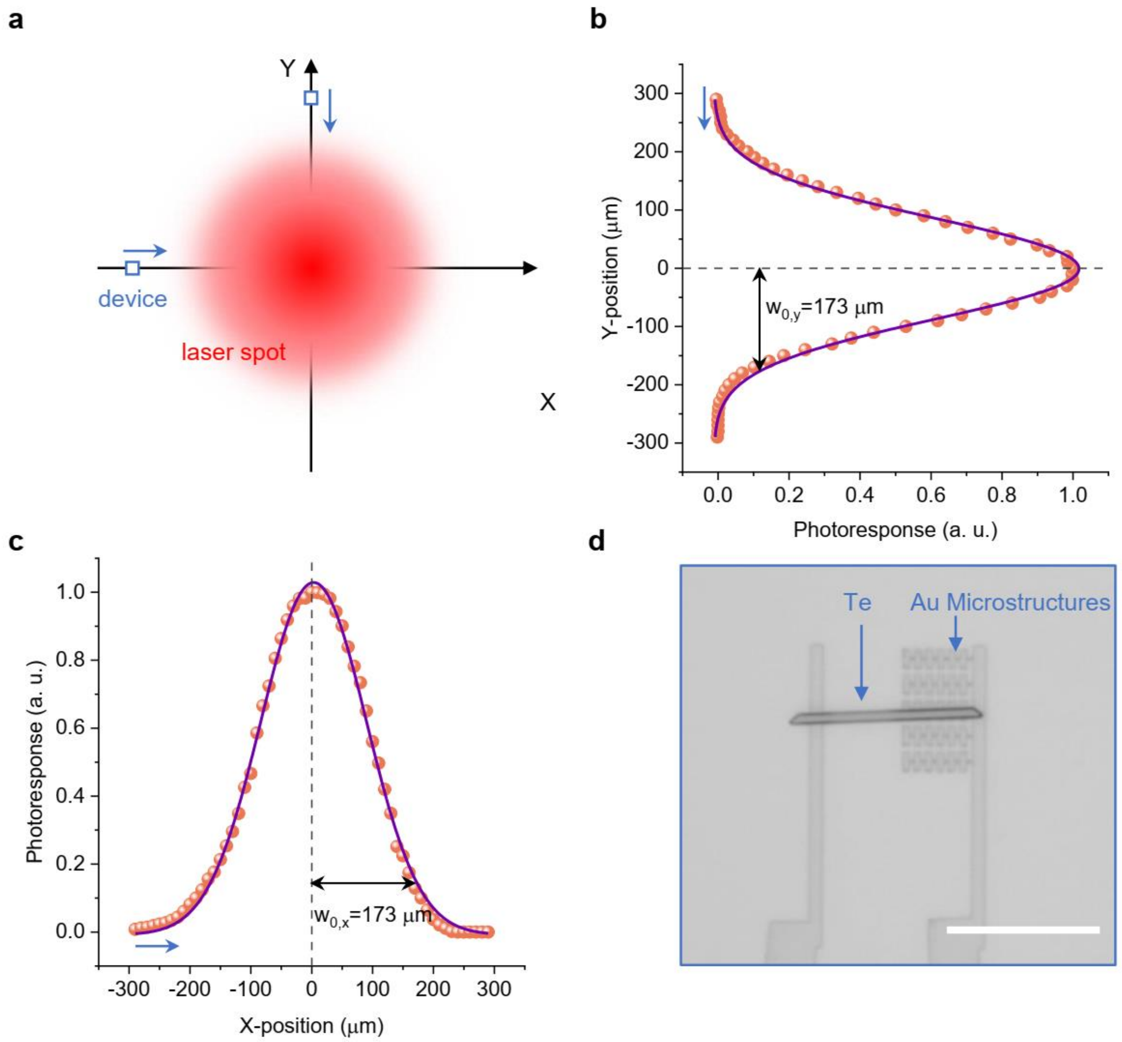


**Supplementary Figure 11: Measurement of the laser spot size**. **a**, Schematic diagram of the laser spot size measurement by scanning our device along X and Y axis, respectively. The blue arrows indicate the device moving directions. **b**, **c**, The experimental photoresponse distributions along Y (**b**) and X (**c**) axis, showing two axis radii (1/$e^2$ intensity) as 173 and 173 µm. Dots are measured data, and lines are fitting curves. **d**, The optical image of the device used for laser spot size measurement. Scale bar: 20 µm.

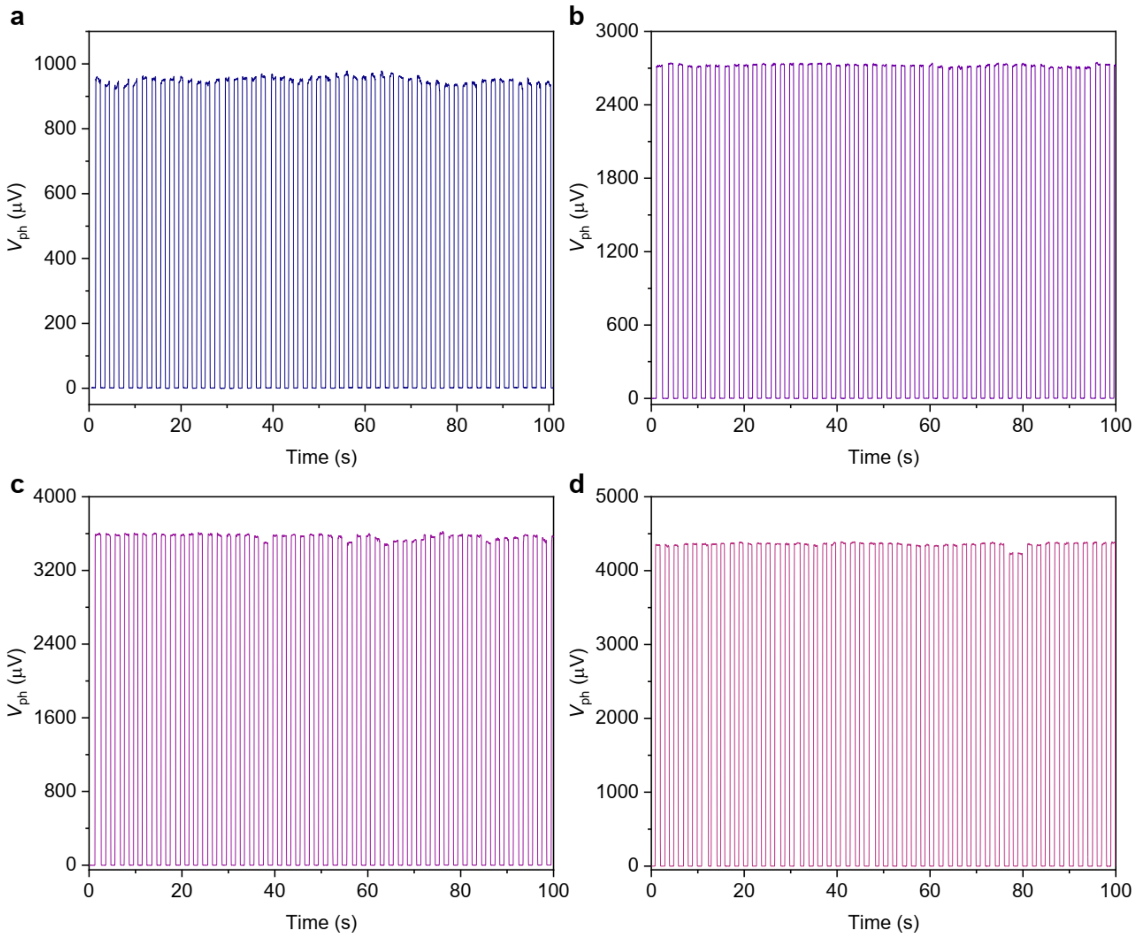


**Supplementary Figure 12: Measured time-resolved photoresponse response of our device with different incident light powers at device**. **a-d,** 2.3 μW (**a**), 6.5 μW (**b**), 8.8 μW (**c**), and 10.6 μW (**d**).

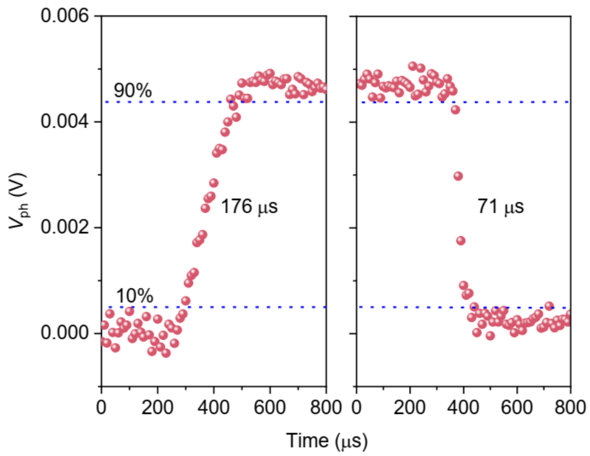


**Supplementary Figure 13: Transient photonresponse of the device.** The transient photoresponse shows a rise time of 176 μs and a decay time of 71 μs, indicating a -3dB bandwidth of 5.7 kHz.

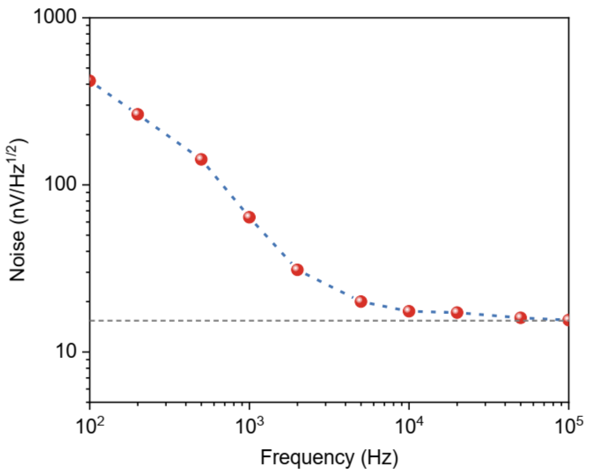


**Supplementary Figure 14: The spectral voltage noise density of the device.** It shows a dramatically decrease with frequency at low frequency range and then keeping a constant when the frequency is over around 5 kHz. The gray dash line indicates the white noise of the device.

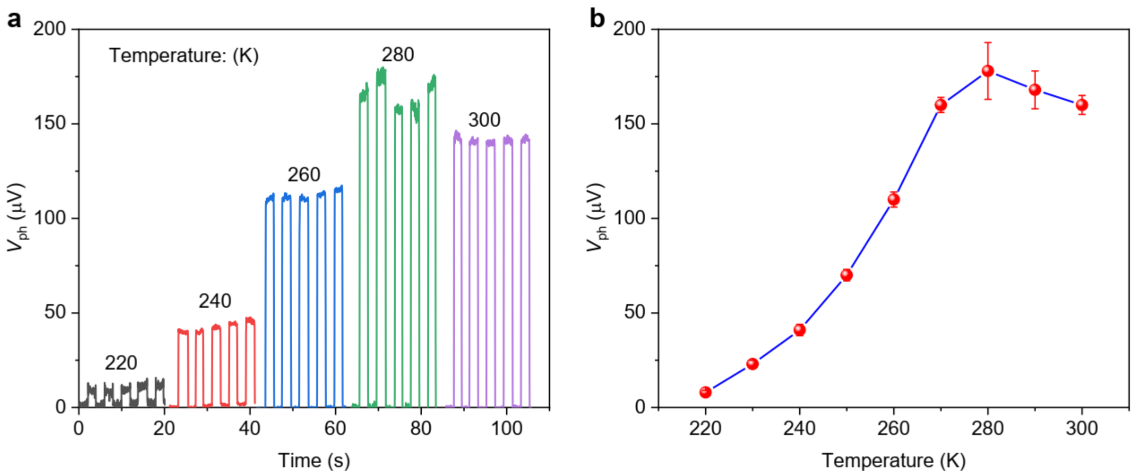


**Supplementary Figure 15: The temperature dependent photovoltage response of a typical device. a**, The photovoltage responses at different temperature. **b**, Temperature dependent photovoltage responses showing that, at a lower temperature, the device exhibits a lower photovoltage response. Symbols are measured data, which are presented as mean values ± SD, n = 5 replicated measurements.

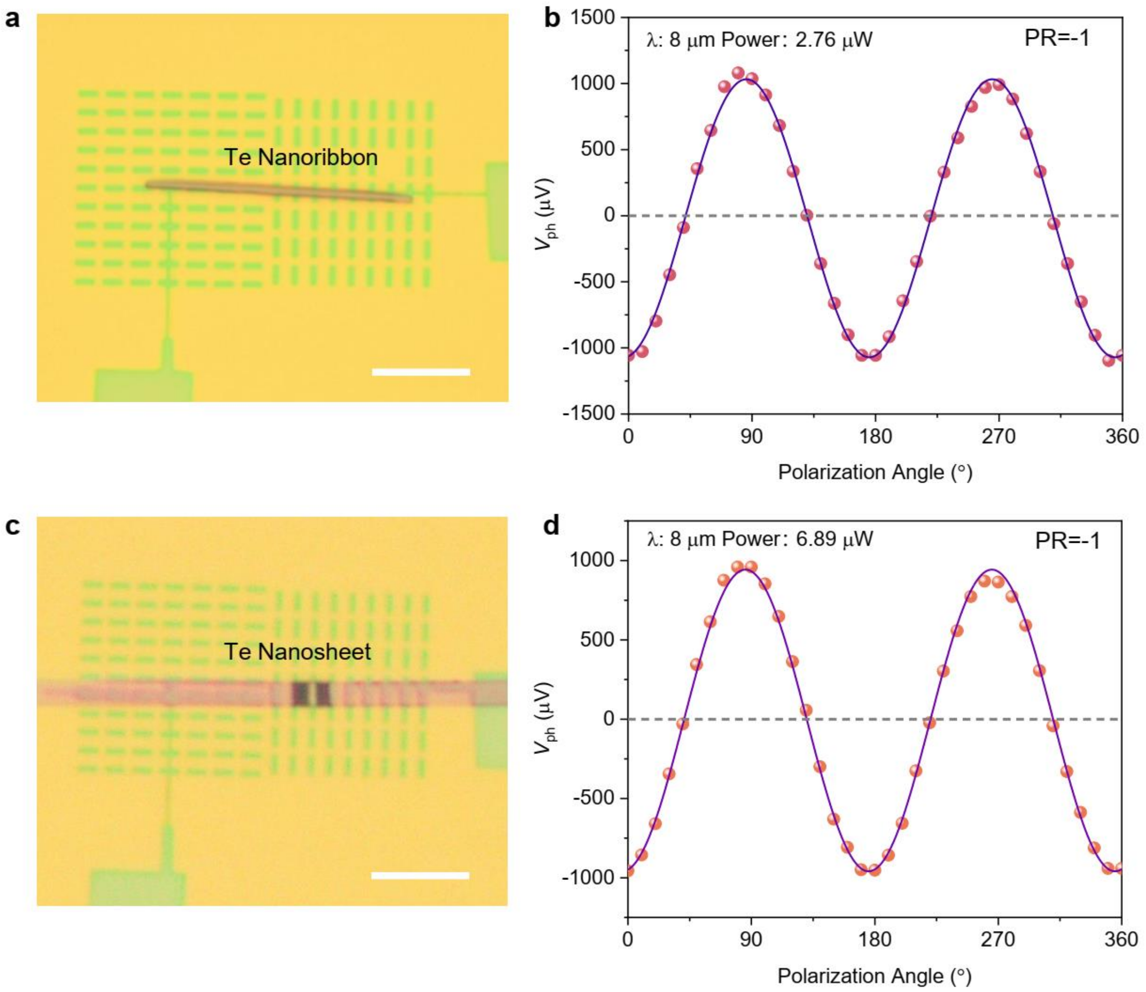


**Supplementary Figure 16: Photoresponses of designed devices with polarization ratio of -1**. **a**, An optical image of the device based on 1D Te nanoribbon and perfect plasmonic absorber array. Scale bar: 10 μm. **b**, The corresponding experimental polarization angle dependent photoresponses showing a bipolar response with a PR=-1. **c**, An optical image of the device based on 2D Te nanosheet and metamaterials. Scale bar: 10 μm. **d**, The corresponding experimental polarization angle dependent photoresponses showing a bipolar response with a PR=-1. Dots are measured data, and lines are fitting curves.

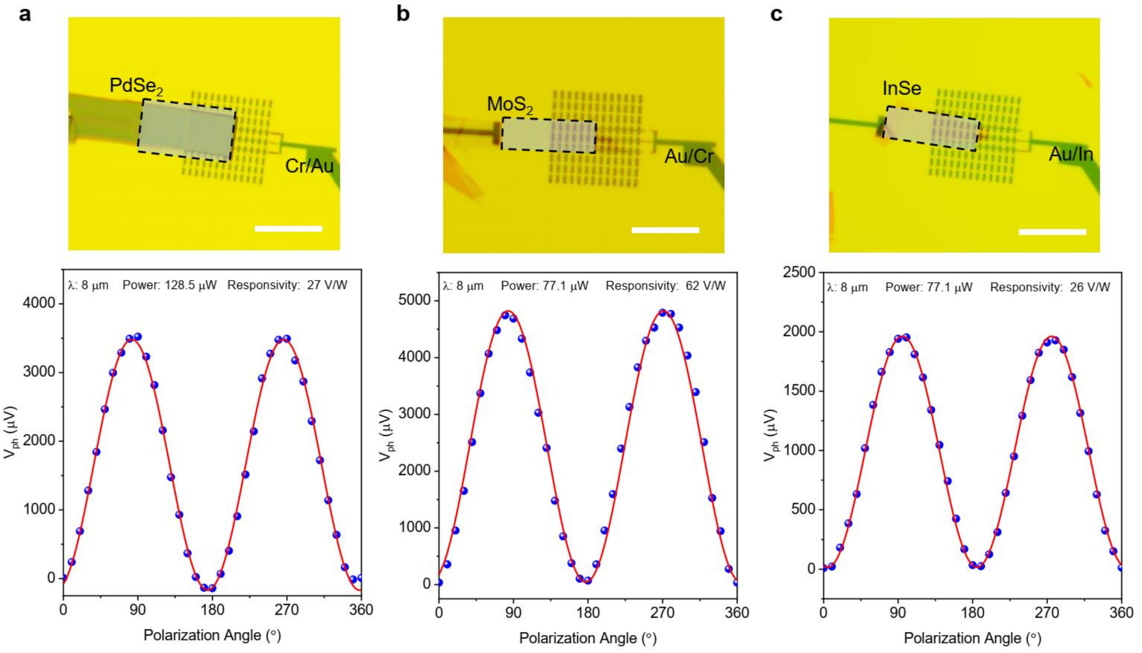


**Supplementary Figure 17: Photoresponses of designed devices with different active 2D materials**. **a**, Optical image and corresponding polarization-sensitive photoresponses of $PdSe_2$ based detector. **b**, Optical image and corresponding polarization-sensitive photoresponses of $MoS_2$ based detector. **c**, Optical image and corresponding polarization-sensitive photoresponses of InSe based detector. The light blue area indicates the optical cross-section area of the devices. Scale bar: 10 μm. Blue dots are measured data, and red lines are fitting curves.

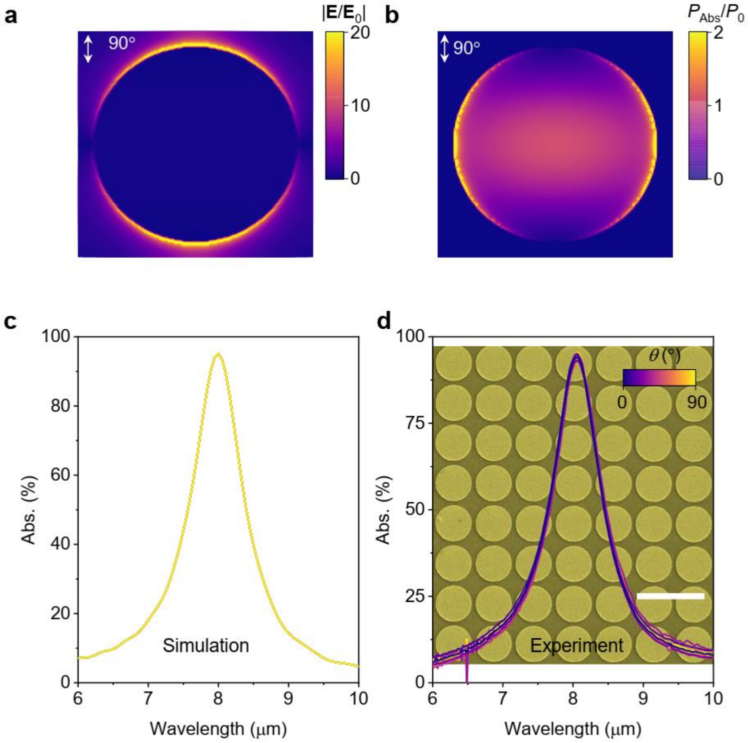


**Supplementary Figure 18: Simulated and experimental measured optical absorption of polarization-insensitive perfect plasmonic absorber $D_1$.** **a**, Full wave simulations of electric field distributions normalized to incident electric field. The period of the gold disk with a radius of 1.3 μm is 3 μm. **b**, Full wave simulations of power absorption density calculated by $P_{abs} = 1/2\omega\varepsilon''|\mathbf{E}|^2$, and normalized by the incident power density $P_0$. **c**, Simulated linear polarization angle dependent absorption spectra. **d**, Experimental measured linear polarization dependent absorption spectra. Inset: SEM image of as-fabricated perfect plasmonic absorber $D_1$. Scale bar 5 μm.

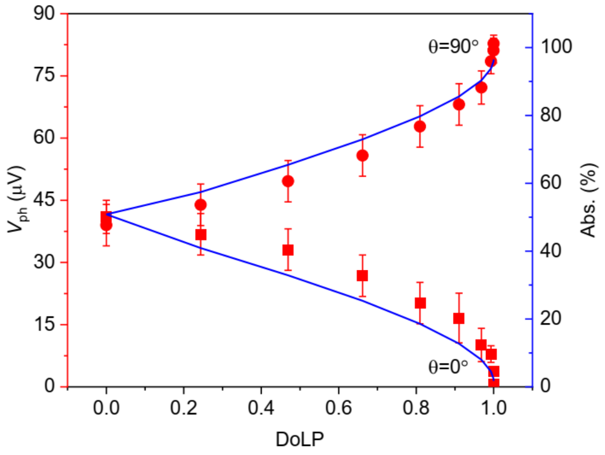


**Supplementary Figure 19: Experimental measured photoresponses and simulated absorption as a function of DoLP of the incident light.** The photoresponses are monotonically increasing (decreasing) with the DoLP increases from 0 to 1 for θ=90° (θ=0°), indicating the ability to detect the DoLP of the incident light. Lines are simulated absorption. Symbols are measured data, which are presented as mean values ± SD, n = 4 replicated measurements.

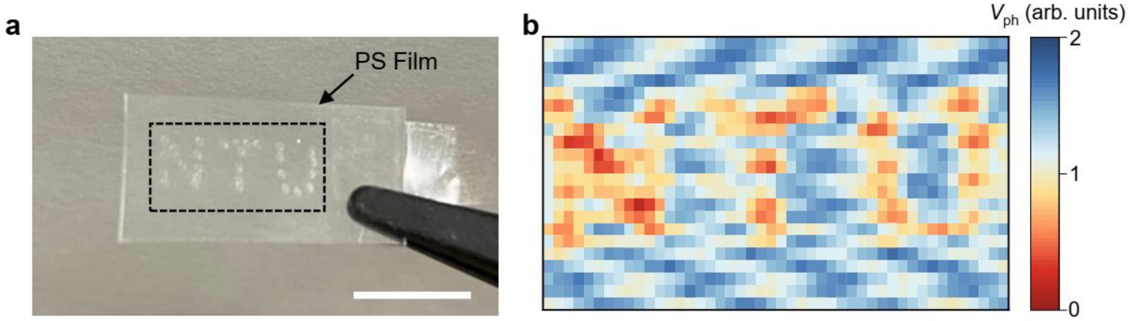


**Supplementary Figure 20: Optical strain measurement for a polystyrene (PS) film with local strain. a**, A typical image of the PS film with local positive strain along with the letter of NTU. Scale bar 0.5 cm. **b**, Experimental measurements of photovoltage response distribution for polarized infrared light transmitted the PS film with local positive strain.

**Supplementary Table 1: Structure parameters of perfect plasmonic absorbers with different resonance wavelengths.**

| | Resonant Wavelength (μm) | $P_1$ (μm) | $P_2$ (μm) | T (μm) | W (μm) | L (μm) |
|---|---|---|---|---|---|---|
| $R_1$ | 7.0 | 2.3 | 3.4 | 0.05 | 0.75 | 2.15 |
| $R_2$ | 8.0 | 2.3 | 3.4 | 0.05 | 0.75 | 2.45 |
| $R_3$ | 9.0 | 2.3 | 3.4 | 0.05 | 0.75 | 2.95 |
| $D_1$ | 8.0 | 3.0 | 3.0 | 0.05 | D=2.6 | |

## Supplementary Table 2. Performance comparison of linear polarization sensitive photodetectors operating in mid-/long-wave infrared region

| Materials | Mechanism | Wavelength (μm) | PR | Response time (s) | Responsivity (V/W) | Detectivity (Jones) | PAS (V/W•degree) | PAD (Jones/degree) | Ref. |
|---|---|---|---|---|---|---|---|---|---|
| InAsSb | PVE | 3-10 | / | $3\times10^{-9}$ | 1.9 mA/W | $6.5\times10^{7}$ | / | / | a |
| MCT | PVE | 2-10 | / | $10\times10^{-9}$ | 185 | $2\times10^{8}$ | / | / | b |
| Gr/Au antenna | PTE | 6.6 | 1.5 | $17\times10^{-9}$ | 92 | / | 0.53 | / | 34 |
| Gr/Au antenna | PTE | 4.0 | →∞ | $<667\times10^{-9}$ | 15.6 | $1.56\times10^{6}$ | 0.27 | $2.7\times10^{4}$ | 8 |
| Gr/Au antenna | BPVE | 4.0 | -1 | / | 27 | $5\times10^{6}$ | 0.94 | $1.7\times10^{5}$ | 31 |
| Twist Gr | BPVE | 7.7 | -2 | $<100\times10^{-6}$ | 3.7 | / | 0.09 | / | 33 |
| $PdSe_2$/Au metamaterials | PTE | 5.3 | →∞ | $76\times10^{-6}$ | 3.6 | $2.5\times10^{5}$ | 0.06 | $4.1\times10^{3}$ | 7 |
| $PdSe_2$ | PTE | 4.6<br>10.5 | 2.03<br>1.21 | $52\times10^{-6}$ | 21.6<br>14.3 | $6.7\times10^{6}$<br>$4.5\times10^{6}$ | 0.19<br>0.04 | $5.9\times10^{4}$<br>$1.2\times10^{4}$ | 16 |
| $TaIrTe_4$ | Shift Current | 4.0 | 8.5 | / | 53.3 | / | 0.82 | / | 32 |
| Te/Au metamaterials | PTE | 8.0 | $2.5\times10^{4}$<br>-1 | $176\times10^{-6}$ | 410<br>380 | $1.7\times10^{7}$<br>$1.1\times10^{7}$ | 7.1<br>13.2 | $2.9\times10^{5}$<br>$3.8\times10^{5}$ | This work |

PVE: photovoltaic effect. PTE: photothermoelectric, BPVE: bulk photovoltaic effect.

PAS: polarization angle sensitivity. PAD: polarization angle detectivity.

a: P13894-011MA from HAMAMASTU. b: PDAVJ10 from THORLABS.

Ref. is cited in the main article.
